# Absence of Bornavirus RNA in Wild Canids and Mustelids in Croatia

**DOI:** 10.3390/microorganisms14040876

**Published:** 2026-04-13

**Authors:** Andreja Jungić, Jelena Prpić, Antun Beljan, Marina Prišlin Šimac, Dinko Novosel, Šimun Naletilić, Marica Lolić, Iva Kilvain, Tibor Andreanszky, Vladimir Savić, Lorena Jemeršić, Mario Škrivanko, Ivana Lojkić

**Affiliations:** 1 Department of Virology, Croatian Veterinary Institute, 10000 Zagreb, Croatia; beljan@veinst.hr (A.B.); prislin@veinst.hr (M.P.Š.); jemersic@veinst.hr (L.J.); ilojkic@veinst.hr (I.L.); 2 Department of Pathology, Croatian Veterinary Institute, 10000 Zagreb, Croatia; dinko.novosel@gmail.com (D.N.); naletilic@veinst.hr (Š.N.); 3 Veterinary Department Vinkovci, Croatian Veterinary Institute, 32100 Vinkovci, Croatia; lolic@veinst.hr (M.L.); skrivanko@veinst.hr (M.Š.); 4 Veterinary Department Rijeka, Croatian Veterinary Institute, 51000 Rijeka, Croatia; kilvain.vzr@veinst.hr (I.K.); andreanszky.vzr@veinst.hr (T.A.); 5 Poultry Center, Croatian Veterinary Institute, 10000 Zagreb, Croatia; v_savic@veinst.hr

**Keywords:** bornavirus, wild canids, mustelids, molecular screening, encephalitis, wildlife surveillance, Croatia

## Abstract

Bornaviruses are neurotropic, negative-sense RNA viruses with zoonotic potential, notably Borna disease virus 1 (BoDV-1) and variegated squirrel bornavirus 1 (VSBV-1). BoDV-1 is endemic in certain regions of Central Europe and maintained in bicolored white-toothed shrews, but its presence in Croatia has not been investigated. Given Croatia’s diverse biogeography and the prevalence of rodent-borne diseases, this study aimed to investigate the presence of orthobornaviruses in wild canids and mustelids. Brain samples from red foxes (*Vulpes vulpes*), golden jackals (*Canis aureus moreoticus*), wolves (*Canis lupus*), martens (*Martes martes*) and badgers (*Meles meles*) were analysed using pan-bornavirus RT-PCR. Despite successful RNA extraction and internal control amplification, bornavirus RNA was not detected in any of the 860 animal samples tested. Although no orthobornavirus RNA was detected, the results still provide valuable information: bornavirus infections appear to be absent or extremely rare in Croatian wild canids and mustelids. By excluding these species as current potential reservoir hosts, this study helps to refine the geographical extent of bornavirus endemicity and emphasises the importance of continuous One Health surveillance in regions with favourable ecological conditions for zoonotic spillover.

## 1. Introduction

Bornaviruses belong to the family *Bornaviridae* within the order *Mononegavirales* [1]. The family currently includes three genera: *Carbovirus*, *Cultervirus*, and *Orthobornavirus* [2,3]. They are enveloped, non-segmented, negative-sense RNA viruses that replicate in the nucleus and exhibit a unique tropism for neural tissue, with a genome of approximately 9 kb [4,5,6]. Members of the genus *Orthobornavirus* infect mammals, birds, and snakes and typically cause persistent, non-cytolytic infections in neurons. The mammalian orthobornaviruses, Borna disease virus 1 and 2 (BoDV-1 and BoDV-2; species *Orthobornavirus bornaense*) and variegated squirrel bornavirus 1 (VSBV-1; species *Orthobornavirus sciuri*), are more closely related to avian orthobornaviruses (ABVs) than to snake orthobornaviruses.

BoDV-1, the causative agent of Borna disease (BD), is maintained in bicolored white-toothed shrews (*Crocidura leucodon*) in parts of Central Europe and can spill over to horses, sheep, humans, and other susceptible species, causing severe encephalitis [7]. The bicolored white-toothed shrew, the only known reservoir host for BoDV-1, is distributed throughout Europe and western Asia and is present in Croatia [8]. Shrews spread BoDV-1 subclinically and excrete the virus in urine, faeces and saliva. Horses, sheep, and humans serve as dead-end hosts and develop severe neurological disorders upon spillover.

VSBV-1 circulates in exotic squirrel species kept as pets and has caused fatal human encephalitis in breeders in Germany [9,10,11]. Thus, BoDV-1 and VSBV-1 are currently the only known bornaviruses with zoonotic potential. The zoonotic risk, combined with their marked neurotropism, which leads to severe and often fatal encephalitis in humans, emphasises their significance for public health.

Although Croatia harbours reservoir-capable animal species such as *C. leucodon*, no systematic surveillance of bornaviruses in wild mammals has been conducted. This knowledge gap hinders accurate risk assessment of zoonotic spillover in south-eastern Europe [7,11]. BoDV-1 is known to be endemic in parts of eastern and southern Germany, eastern Switzerland, Liechtenstein and the Austrian federal states of Upper Austria and Vorarlberg [7,12,13]. Given the recent increase in reports of bornavirus-associated encephalitis in Europe, establishing baseline data from non-endemic regions such as Croatia is increasingly important for understanding the broader epidemiology and potential southward spread of orthobornaviruses.

Croatia, situated at the crossroads of Central Europe, Southeast Europe and the Mediterranean, has rich biogeographical diversity. The Croatian Adriatic coast is characterised by a Mediterranean climate that favours diseases transmitted by insect vectors, while the continental climate of the mainland is favourable for viruses transmitted by rodents. Some of these viruses are endemic, while others are emerging, such as hantavirus [14], lymphocytic choriomeningitis virus (LCMV) [15] and hepatitis E virus (HEV) [16]. Croatia’s varied geography, ranging from lowland forests and karst fields to mountainous terrain, supports a diverse range of wildlife, including red foxes (*Vulpes vulpes*), golden jackals (*Canis aureus moreoticus*) and mustelids. These species are widely distributed throughout the country and often inhabit areas near human settlements, agricultural zones and rodent-rich environments [17]. Their ecological behaviours, such as scavenging and predation on small mammals, increase the likelihood of contact with potential reservoir hosts such as shrews and rodents. In particular, the expansion of golden jackal and red fox populations in Croatia has been linked to land use and climate change, which could further favour the spread of zoonoses [18]. Therefore, wild canids could be valuable sentinel species for emerging zoonoses, including bornaviruses.

To account for the great diversity of bornaviruses and their potential hosts, we used a pan-bornavirus RT-qPCR validated method for detecting several bornavirus genera in different species [2]. This method enables screening in ecosystems where host–virus relationships remain poorly understood and is required to ensure detection across divergent viral lineages and potential hosts. We hypothesised that wild canids and mustelids in Croatia could harbour orthobornaviruses and serve as accidental hosts, given their ecological overlap with established reservoir species and their scavenging habits.

Bornavirus research in Croatia has been minimal, with only a single detection of VSBV-1 in a captive squirrel reported to date [11]. Although reservoir hosts are present and environmental conditions support the circulation of rodent-borne pathogens, orthobornavirus surveillance in the country has not been systematically undertaken. This study therefore provides the first structured investigation of orthobornaviruses in free-living canids and mustelids in Croatia. By extending surveillance beyond known endemic regions, it offers new insights into potential silent circulation in south-eastern Europe and contributes essential baseline data for understanding virus ecology in non-endemic settings.

As Croatia is not located in a BoDV-1 endemic area, the aim of this study was to investigate the presence of orthobornaviruses in a population of wild canids. In this study, conducted between 2023 and 2025, we analysed brain samples from red foxes (*V. vulpes*), golden jackals (*C. aureus moreoticus*), mustelids (martens (*M. martes*) and badgers (*M. meles*)) from different regions of Croatia. These species were selected based on their population density, ecological behaviour, and likelihood of contact with rodentreservoirs.

## 2. Materials and Methods

**Figure 1:**
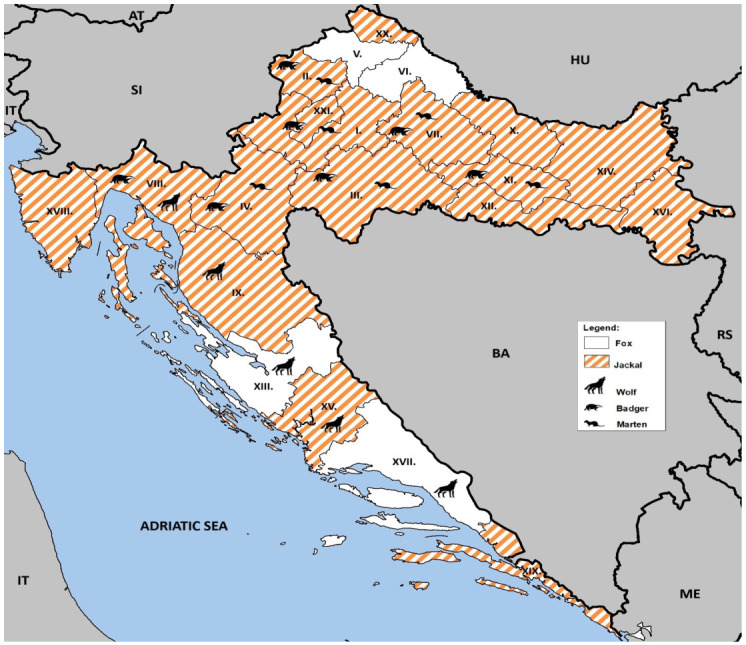
Map of Croatia indicating the counties where the red fox, jackal and mustelid samples were collected. On the map, counties are denoted by Roman numerals (I–XXI) as follows: I. Zagreb County, II. Krapina–Zagorje, III. Sisak–Moslavina, IV. Karlovac, V. Varaždin, VI. Koprivnica–Križevci, VII. Bjelovar–Bilogora, VIII. Primorje–Gorski Kotar, IX. Lika–Senj, X. Virovitica–Podravina, XI. Požega–Slavonia, XII. Brod–Posavina, XIII. Zadar County, XIV. Osijek–Baranja, XV. Šibenik–Knin, XVI. Vukovar–Srijem, XVII. Split–Dalmatia, XVIII. Istria, XIX. Dubrovnik–Neretva, XX. Međimurje, XXI. The City of Zagreb County. Countries with international borders to Croatia are Bosnia and Herzegovina (BA), Hungary (HU), Montenegro (ME), Serbia (RS), and Slovenia (SI); Croatia shares a maritime border with Italy (IT) in the Adriatic Sea.

Brain samples from red foxes, jackals, and mustelids were collected between December 2023 and March 2025 as part of a national rabies surveillance program prescribed by the Croatian Ministry of Agriculture, Veterinary and Food Safety Directorate. Samples were selected based on their quality and geographical origin (Figure 1).

Animals in this study were submitted to the Croatian Veterinary Institute primarily for rabies virus (RABV) testing as part of passive and active monitoring. Brain samples were collected as whole-brain tissue from carcasses into polypropylene containers (safety screw-top container, 120 mL, DeltaLab, Rubi, Barcelona, Spain) by trained pathology staff. Brain samples were selected from the national rabies surveillance pool based on sample integrity, availability of complete brain tissue, and broad geographical distribution to ensure representation of all major Croatian regions. Poor quality samples or those showing signs of autolytic processes were excluded from further processing. For the analysis, the central part of the brain was sampled, including sections of the frontal, temporal, and parietal lobes, as well as the olfactory region and hippocampus. The hippocampus region was chosen as the primary diagnostic matrix because orthobornaviruses exhibit strong neurotropism, and the central nervous system is the site where viral RNA is most consistently detected during infection. If RNA extraction was not carried out immediately, the brain samples were stored at −20 °C.

The samples were first analysed for the presence of RABV using the direct fluorescent antibody (DFA) test [19] and/or the RT-qPCR assay for pan-lyssavirus LN34 [20], then stored at −80 °C until further analysis. The approximate dental age of the foxes and jackals was determined by microscopy for the purpose of the rabies oral vaccination monitoring [21].

For RNA extraction, brain samples were homogenised in phosphate-buffered saline (PBS), pH 7.4, to obtain 10% (*w*/*v*) brain suspensions. These suspensions were centrifuged at 220× *g* for 10 min and then used as starting material for RNA extraction. To monitor RNA extraction and detect potential PCR inhibitors, samples were also analysed for the presence of the mammalian beta-actin gene [22]. Viral RNA was extracted from 200 μL of the supernatant of the centrifuged suspensions using a MagMAX Core Kit (Thermo Fisher Scientific, Waltham, MA, USA) on a KingFisher Flex System (Thermo Fisher Scientific, Waltham, MA, USA), according to the manufacturer’s instructions. The RNA extracts were stored at −80 °C until use. In total, 624 red foxes, 196 golden jackals, 19 martens, 14 badgers and seven wolves were analysed for bornavirus RNA (Table 1). This represents one of the most comprehensive data sets for wild canids analysed in south-eastern Europe.

To detect a broad spectrum of orthobornaviruses, including BoDV-1 and BoDV-2, we used panBorna mix 7.2 as described [23]. RT-qPCR was performed using AgPath-ID™ One-Step RT-PCR reagents (Thermo Fisher Scientific, Waltham, MA, USA). The panBorna mix 7.2 assay was selected because it detects a wide range of orthobornaviruses, including BoDV-1, BoDV-2, and VSBV-1, with high analytical sensitivity and specificity. This assay has been validated for wildlife screening and is suitable for detecting both mammalian and avian bornaviruses. Although broad-range PCRs may show reduced specificity, the pan-Borna mix 7.2 assay retains a low limit of detection and can detect very small amounts of viral RNA. Each reaction contained 0.5 μL enzyme mix, 6.25 μL reaction mix, 0.94 μL of each primer (10 pmol/μL), 0.32 μL probe (10 pmol/μL), 1.25 μL beta-actin mix (2.5 pmol/μL primer and probe), 0.3 μL RNase-free water, and 2.0 μL RNA template or NTC, in a total volume of 12.5 μL. With AgPath-ID™ One-Step RT-PCR reagents (Thermo Fisher Scientific, Waltham, MA, USA), the thermal profile consisted of one cycle at 45 °C for 10 min and 95 °C for 10 min, followed by 50 cycles at 95 °C for 15 s and 60 °C for 45 s. All RT-qPCRs were performed using the CFX96 Opus Real-Time PCR Detection System (Bio-Rad Laboratories, Hercules, CA, USA) and CFX Maestro v2.3 software.

RNA from avian bornavirus-positive chicken brain tissue, kindly provided by Prof Željko Gottstein (Faculty of Veterinary Medicine, University of Zagreb, Croatia), was used as a positive control. Aliquots of ultrapure water were used as negative controls. To minimize the risk of PCR contamination, standard precautions were implemented, including the use of a closed system for amplification and detection. The results were expressed as cycle-of-quantification (Cq) values.

## 3. Results

All brain samples tested with the DFA test were negative for rabies antigen, and RT-qPCR was negative for the rabies viral genome.

RNA extraction was successful in all samples, as confirmed by consistent amplification of the internal beta-actin control, indicating no significant PCR inhibition. However, orthobornavirus RNA was not detected in any of the 860 animals tested (Table 1 and Figure 1).

These negative results were consistent across animal species, geographical regions, age groups and collection seasons, indicating that no evidence of detectable viral RNA, i.e., infection with ongoing viral replication at the time of sampling, was found in wild canids and mustelids sampled in Croatia during the study period. Given the sample size of 860 animals, the study was expected, with 95% probability, to detect infections occurring at a prevalence of approximately 0.35% or higher. In practical terms, this means that if orthobornavirus were circulating at moderate prevalence (≥3–4 infected animals per 1000), at least one positive case would be expected. The absence of detections therefore suggests that, if present, orthobornavirus circulation in these species is likely to occur at very low (<0.3%) or sporadic levels.

## 4. Discussion

In this study, we screened free-living red foxes, golden jackals, and a limited number of wolves and mustelids across Croatia for evidence of orthobornavirus infection. Confirmed orthobornavirus infections, specifically those with BoDV-1, are restricted to relatively small areas of Central Europe, which may partly reflect rare spillover events and the sporadic nature of outbreaks. However, seroepizootiological studies have shown that BoDV-1-specific antibodies have been detected in horses, cattle, and sheep in an increasing number of countries within and outside Europe [24,25,26]. The bicolored white-toothed shrew, the only known reservoir host of BoDV-1, can infect other non-reservoir mammals through spillover transmission, causing neurological signs [27]. The various clinical manifestations of Borna disease result not from direct viral damage but from the host’s immune response to the virus, which triggers cell-mediated immunopathological reactions leading to persistent central nervous system infection and immune-mediated encephalomyelitis [28].

Although the incidence appears to be rare, single documented cases of BoDV-1 infection in a dog and a cat [29,30] suggest that carnivores such as wild canids may be susceptible to infection and disease. Accidental BoDV-1 infections have been reported in several domestic species, most notably horses, alpacas and other livestock, which are affected more frequently than foxes and can serve as valuable sentinel hosts in endemic areas [13,26]. In Croatia, however, routine post-mortem sampling of these species is limited, whereas red foxes are consistently collected through the national rabies surveillance program. This long-standing and well-structured sampling effort provides a rare opportunity to screen a large number of CNS tissues and to explore potential bornavirus circulation in wildlife. For this initial assessment, foxes therefore represented the most practical and accessible study population. Still, targeted surveillance in clinically affected domestic species, particularly horses, would be an important next step to complement wildlife data and strengthen early detection of spillover events.

Red foxes are reservoirs for several pathogens of veterinary and public health significance [5]. In Europe, canine distemper virus (CDV) is the most frequently detected pathogen in red foxes with non-suppurative meningoencephalitis [31], while other viral causes of encephalitis include rabies lyssavirus [32], Aujeszky’s disease virus (SHV-1) [33,34], canine adenovirus type 1 [35], and canine circovirus [36]. CDV and canine circovirus have already been documented in Croatian foxes [37,38]. In addition, foxes can be carriers of zoonotically important pathogens such as *Echinococcus multilocularis* [39], which has also been found in Croatian red foxes [40].

Foxes may be exposed to bornaviruses when they inhabit environments where infected reservoir hosts are present, particularly through scavenging or predation [41]. They are opportunistic feeders and adapt their diet to what is available in their environment. Common foods include small mammals such as rodents and rabbits, birds, insects, fruits, and berries. They also eat carrion and often feed in areas where humans live. Badgers and martens also frequently include small rodents in their diet, and foxes and badgers often share their habitat [42]. Golden jackals are omnivores, predators and scavengers and are of particular interest as potential carriers of zoonotic diseases, highlighting the importance of including them in wildlife pathogen surveillance.

According to national estimates, the Croatian populations of red foxes and golden jackals comprise approximately 15,000 and 10,000 animals, respectively, with both the abundance and range of jackals having increased markedly over the past 15 to 20 years. Other animal species routinely sampled within the national rabies surveillance programme were also included in our investigation. To our knowledge, this is the first study to use apparently healthy, shot red foxes and jackals obtained through the oral rabies vaccination (ORV) campaign, implemented in Croatia since 2011 [43], to investigate bornavirus infection.

To date, foxes have been examined for bornaviruses using several methods. Among serological methods, the most common are the indirect immunofluorescence test (IIFT) and immunoblot for detecting reactive bornavirus antibodies in serum [23,44]. As neutralising antibodies are synthesised late after infection, during the chronic phase of the disease [45,46], it is likely that their detection in serum would coincide with the presence of viral RNA in the brain. Consistent with previous studies, we were unable to detect orthobornavirus RNA in the brain samples of the examined animals using RT-qPCR. The absence of evidence for bornavirus infection in foxes is in line with previous studies on foxes in both endemic and non-endemic regions of Germany and Austria [44,47].

We acknowledge that using an avian rather than a mammalian orthobornavirus control is a methodological limitation. Although the avian bornavirus positive control reliably confirms assay performance, it does not fully represent the genetic diversity of mammalian orthobornaviruses. Consequently, minor sequence differences could affect amplification efficiency and reduce sensitivity for detecting divergent mammalian strains in foxes and jackals. Nevertheless, the panBorna 7.2 assay has been validated for a broad spectrum of orthobornaviruses [23], which mitigates, but does not entirely eliminate, this limitation. As the incidence of Borna disease varies seasonally [27], limited study periods can affect detection. However, our sampling extended over almost two years, covering all seasons and age groups, so this potential bias was minimised. As the health status of these animals is unknown, signs of encephalitis cannot be excluded, since no pathological examination was carried out. Brain tissue remains the most important diagnostic target, but relying solely on it may miss infections confined to other organs [48]. Therefore, methodological refinement could improve surveillance outcomes.

Serological surveillance is an important tool for assessing exposure to bornaviruses, especially when RNA detection fails. However, as these methods are time-consuming and labour-intensive, they were not suitable for this type of screening, although their use would increase the reliability of exposure estimates and support broader epidemiological conclusions [49].

Reservoir hosts, such as bicolored white-toothed shrews, were not sampled, limiting conclusions about virus circulation in the primary host population. This was a convenience sampling, and therefore carries intrinsic constraints regarding representativeness and completeness. There is an absence of published ecological or epidemiological data on Crocidura leucodon in Croatia, which prevents placing our findings in a broader local context. Only brain tissue was analysed, although bornavirus RNA may occasionally be detectable in peripheral organs, and no histopathological examination was performed, so subclinical encephalitis could not be assessed. The number of mustelids and wolves was small, reducing statistical power for these species. Finally, passive surveillance may introduce sampling bias towards animals found dead or shot, which may not fully represent the wild population. Nevertheless, testing in regions without recognised endemic foci is important, as it may reveal previously unrecognised bornaviruses in potential new sources of infection and help clarify the geographical limits of bornavirus circulation. There is also the question of possible transmission of avian bornaviruses to wild canids such as red foxes, as seen in the current epidemic of highly pathogenic avian influenza virus. For all these reasons, we used a pan-bornavirus real-time RT-PCR protocol.

Furthermore, we have chosen a passive surveillance approach in which wild canids serve as indicators of possible bornavirus infection in the local small mammal population. Even if no viruses are detected, such data are valuable for refining epidemiological baselines.

Given the large sample size, broad geographical coverage, and multiseasonal design, our results provide strong evidence that bornavirus infections in wild canids in Croatia are currently absent or extremely rare. Furthermore, these findings indicate that spillover to local small mammal populations is currently unlikely.

By 2013, fatal human cases caused by BoDV-1 and cases of VSBV-1-associated encephalitis had already been reported [50]. More recent evidence has strengthened the role of bornaviruses as emerging pathogens causing severe encephalitis in humans. Early detection of bornaviruses in species such as foxes, jackals, and other small mammals could provide advance warning of spillover risks to domestic animals and humans.

Although no orthobornavirus RNA was detected in the examined animals, the results provide an important epidemiological reference point for a region where the circulation of these viruses has not previously been assessed. Bornaviruses are characterised by highly focal endemicity and a strong dependence on reservoir–host ecology, which means that their detection outside known endemic zones requires systematic, long-term monitoring. In this context, wild canids and mustelids represent suitable sentinel species because their feeding behaviour, mobility, and habitat overlap with small mammals create natural opportunities for exposure to potential reservoir hosts. Their inclusion in surveillance programmes therefore offers a practical means of identifying early signs of virus introduction or changes in local pathogen dynamics. The demographic expansion of golden jackals and the stable, widespread presence of red foxes across Croatia further increase the relevance of these species, as ecological shifts can influence the distribution and transmission of wildlife pathogens. Although our findings were uniformly negative, they nonetheless contribute valuable information by narrowing the current geographical boundaries of orthobornavirus activity in south-eastern Europe. Negative surveillance data are often overlooked, yet they are essential for refining risk assessments, guiding targeted sampling strategies, and informing One Health preparedness. The use of a broad-range pan-bornavirus RT qPCR assay strengthens the reliability of our conclusions, as this method is designed to detect diverse orthobornaviruses, including potentially divergent strains. However, the absence of RNA detection does not exclude the possibility of low-level or spatially restricted circulation, particularly in reservoir hosts that were not sampled. Bornaviruses are known to persist in specific microhabitats, and their detection often requires complementary approaches such as serology, reservoir host trapping, and ecological assessments of small mammal communities. Integrating these methods into future studies would provide a more complete understanding of potential virus circulation and help identify environmental or ecological factors that may influence spillover risk. Given the recognition of BoDV 1 and VSBV 1 as causes of severe human encephalitis, establishing baseline data from non-endemic regions remains essential for early detection and risk mitigation. In this broader context, our study provides a foundation for future surveillance efforts and contributes to a more comprehensive understanding of orthobornavirus ecology in regions adjacent to known endemic areas.

## 5. Conclusions

In summary, this study represents the first systematic surveillance of orthobornavirus infection in wild canids and mustelids across Croatia. Despite extensive sampling across regions and seasons, no bornavirus RNA was detected. Although no viral RNA was detected, these findings should be interpreted with caution. The absence of detection does not exclude the possibility of low-level, sporadic, or geographically restricted circulation, particularly given the lack of serological data and the use of an avian positive control. These results suggest that, if orthobornavirus infections occur in these species in Croatia, they are likely rare. While limitations in sampling reservoir hosts may influence detection, wildlife surveillance remains relevant beyond animal health, contributing directly to public health preparedness. Integrating bornavirus surveillance within a One Health framework would facilitate coordinated responses across human, veterinary, and environmental health sectors [51].

Future studies incorporating reservoir hosts such as shrews, as well as serological screening of wildlife and domestic animals, would further strengthen early-warning capacity and help define the true geographical boundaries of bornavirus circulation in Europe.

## Figures and Tables

**Table 1:** The number of animal brain samples tested per county.

County	Red Fox	Golden Jackal	Badger	Marten	Wolf
City of Zagreb and Zagreb County	55	15	1	3	0
Krapina–Zagorje	33	2	2	6	0
Sisak–Moslavina	58	31	3	1	0
Karlovac	67	18	1	3	0
Varaždin	24	0	0	0	0
Koprivnica–Križevci	9	0	0	0	0
Bjelovar–Bilogora	34	2	2	4	0
Primorje–Gorski Kotar	29	6	1	0	2
Lika–Senj	13	10	0	0	1
Virovitica–Podravina	21	6	0	0	0
Požega–Slavonija	31	1	4	2	0
Brod–Posavina	22	37	0	0	0
Zadar	36	0	0	0	1
Osijek–Baranja	43	27	0	0	0
Šibenik–Knin	33	1	0	0	1
Vukovar–Srijem	19	23	0	0	0
Split–Dalmacija	47	0	0	0	2
Istria	28	15	0	0	0
Dubrovnik–Neretva	14	2	0	0	0
Međimurje	8	0	0	0	0
Total	624	196	14	19	7

## Data Availability

The original contributions presented in this study are included in the article. Further inquiries can be directed to the corresponding authors.
